# Assessing the feasibility of test-and-cull and test-and-segregation approaches for the control of high-prevalence bovine tuberculosis in Ethiopian intensive dairy farms

**DOI:** 10.1038/s41598-024-64884-x

**Published:** 2024-06-21

**Authors:** Matios Lakew, Biniam Tadesse, Sreenidhi Srinivasan, Muluken Aschalew, Bekele Andarge, Dirshaye Kebede, Addisu Etifu, Tena Alemu, Bekele Yalew, Teferi Benti, Abebe Olani, Shubisa Abera, Wegene Bedada, Abebe Fromsa, Getnet Abie Mekonnen, Gizat Almaw, Gobena Ameni, Hagos Ashenafi, Balako Gumi, Douwe Bakker, Vivek Kapur

**Affiliations:** 1 Aklilu Lemma Institute of Pathobiology, Addis Ababa University, P.O. Box 1176, Addis Ababa, Ethiopia; 2 Animal Health Institute, P.O. Box 04, Sebeta, Ethiopia; 3 Global Health Initiative, Henry Ford Health, Detroit, MI 48202 USA; 4 Livestock Development Institute, Bishoftu, Ethiopia; 5 Wolkite University, Gubre, Ethiopia; 6 Department of Veterinary Medicine, College of Agriculture and Veterinary Medicine, United Arab Emirates University, PO Box 15551, Al Ain, United Arab Emirates; 7 Independent Researcher and Technical Consultant, Lelystad, The Netherlands; 8 Departamento de Sanidad Animal, Facultad de Veterinaria, Universidad Complutense, Madrid, Spain; 9 Department of Animal Science, The Pennsylvania State University, University Park, PA USA; 10 Huck Institutes of the Life Sciences, The Pennsylvania State University, University Park, PA USA

**Keywords:** Bovine tuberculosis, Disease control, Test-and-cull, Test-and-segregation, Diagnostic thresholds, Economic implications, Microbiology, Diseases

## Abstract

Bovine tuberculosis (bTB) is endemic and has a substantial impact on the livestock sector in Ethiopia and other low and middle-income countries (LMICs). With a national emphasis on dairy farm intensification to boost milk production and spur economic growth, the incidence of bTB is anticipated to rise. However, Ethiopia, like other LMICs, lacks a comprehensive national bTB control strategy due to the economic and social infeasibility of traditional test-and-cull (TC) approaches. To inform the development of such a strategy, we evaluated the effectiveness and feasibility of TC and test-and-segregation (TSg) strategies for bTB control on Ethiopian dairy farms. A TC approach was used at Farm A [N = 62; comparative cervical test (CCT) > 4 mm, starting prevalence 11.3%] while TSg was implemented at Farm B (N = 45; CCT > 4 mm, prevalence 22.2%), with testing intervals of 2–4 months. Both strategies achieved a reduction in bTB prevalence to 0%, requiring seven rounds of TC over 18 months at Farm A, and five rounds of TSg over 12 months at Farm B’s negative herd. The results show that adopting more sensitive thresholds [CCT > 0 mm or single cervical test (SCT) > 2 mm] during later rounds was pivotal in identifying and managing previously undetected infections, emphasizing the critical need for optimized diagnostic thresholds. Cost analysis revealed that TC was approximately twice as expensive as TSg, primarily due to testing, labor, and cow losses in TC, versus construction of new facilities and additional labor for TSg. This underscores the economic and logistical challenges of bTB management in resource-limited settings. Taken together, our study highlights an urgent need for the exploration of alternative approaches including TSg and or vaccination to mitigate within herd transmission and enable implementation of bTB control in regions where TC is not feasible.

## Introduction

Bovine tuberculosis (bTB) is a chronic infectious disease of cattle, other domesticated animals, and certain wildlife populations caused by members of the *Mycobacterium tuberculosis* complex (MTBC). Zoonotic tuberculosis resulting from transmission to humans constitutes a public health problem^1^. A number of highincome countries eradicated bTB from their cattle population using a test-and-cull (TC) method, consisting of repeated intradermal tuberculin testing and subsequent culling of reactor animals^2–4^. In addition, the public health impact of bTB was also reduced in most countries that had not yet implemented the bTB eradication programmes by the introduction of better food safety standards such as the pasteurization of milk.

Nevertheless, the public health and economic impacts of bTB remain in most low-and-middle income countries (LMICs) where the disease control and eradication programs are not yet in place/fully implemented. In 2019, the World Health Organization (WHO) estimated a global occurrence of approximately 140,000 (range of 69,800–235,00) new cases of zoonotic tuberculosis (TB) caused by *Mycobacterium bovis*^5^*.* This accounted for 1.4% of the overall global TB burden and the majority of these cases were documented in low- and middle-income countries (LMICs). Additionally, a recent study in Central Ethiopia revealed a prevalence of 2.4% for zoonotic TB^6^.

The conventional approaches employed to control bTB in cattle population include TC and test-and-segregation (TSg). TC, a widely adopted approach for bTB elimination, involves slaughtering animals that test positive for the disease. A notable example is the United States’ highly successful campaign to eliminate bTB, during which approximately 232 million cattle were tested between 1917 and 1940, leading to the culling of 3.8 million reactor cattle^3^. The campaign resulted in a dramatic reduction in human deaths and suffering, and conferred substantial benefits to the farm sector, estimated to be roughly ten times the costs, which exceeded $3 billion (in year 2000 dollars) by 1962. This underscored the substantial investment needed to curb the disease and also the significant economic and health benefits that accrued from implementing effective bTB control strategies.

As an alternative to TC, TSg approach, also known as the “Bang” method, was employed by several countries during the earlier years of bTB control^7,8^. This method primarily involves the segregation of bTB positive animals from the herd through successive testing, thereby establishing a bTB-test negative herd. In this approach, segregated positive animals are kept under close monitoring and removed only when they show clinical signs. However, it’s crucial to have a comprehensive understanding of how both control options perform under local conditions to identify the most effective strategy for bTB control in LMICs.

The tuberculin skin test (TST) is the standard test recommended for the diagnosis of bTB in live cattle^1^. TST can be either the single intradermal test (SIT) or the comparative cervical test (CCT). In the SIT, the bovine tuberculin purified protein derivative (PPDB) is injected, either in the neck area for the single cervical test (SCT) or at the base of the tail for the caudal fold test (CFT). The CCT test requires simultaneous injection of both avian and bovine PPD in the neck region. Crucially, effective bTB control necessitates the selection of most suitable TST and determining the appropriate interpretation of the test results.

Despite bTB being a top-priority livestock and zoonotic disease in Ethiopia, a national strategy for its control has not yet been developed and implemented^9,10^. Pilot studies using test-and-segregation showed a reduction in bTB prevalence from 48 to 1% after four rounds of testing^11^. Likewise, another study implementing the test-and-cull method showed that a farm reached a test-negative status earlier using a CCT > 4 mm cut-off^12^. Nevertheless, the application of more sensitive tests revealed the presence of test-positive animals in the herd during the final rounds. This highlights the necessity of employing a more sensitive test interpretation than the CCT > 4 mm cut-off to remove the last remaining bTB-infected animals from the herd. To achieve this, a thorough characterization of available diagnostic tests and their respective cut-offs is essential.

Therefore, the primary aim of this study was to assess the effectiveness and feasibility of implementing test-and-cull and test-and-segregation approaches on a pilot scale in intensively raised dairy cattle settings in Ethiopia. Additionally, the second objective was to gain insights into the diagnostic thresholds of the current intradermal skin tests for effective bTB control, tailored to the Ethiopian context and potentially applicable in other LMICs.

## Materials and methods

### Dairy farms

The study was conducted on two dairy farms, here designated as Farm A and Farm B, in the central dairy milk shed of Ethiopia. Both dairy farms, owned by the government, were selected for the study due to the interest to control bTB to improve the overall health and productivity of the farms, as part of a larger economic development initiative to ensure self-sufficiency in livestock products and for the creation of new markets for the livestock industry. The dairy farms possessed the necessary space, infrastructure, and trained personnel supervised by veterinarians to effectively manage bTB-positive and negative animals separately while conducting bTB control activities. Both farms have an intensive and closed management system: no cattle are purchased from other farms, with artificial insemination used for breeding and herd propagation. During the bTB control efforts on Farm A, male animals along with some heifers were transferred to other facilities under contract or sold, while Farm B did not sell any animals. All bTB control activities were conducted during a period of 18 months from January 2022 to August 2023 on Farm A. Initially, 62 animals (47 females and 15 males) were tested in this farm. On Farm B, the bTB control measures were carried out over a 12-month period, extending from August 2022 to August 2023 with 45 female animals tested during the first round.

### Study design

A repeated cross-sectional study was conducted on both farms. At Farm A, a TC approach was used to control bTB. The farm was tested for seven consecutive rounds at 2–4 months intervals, considering the desensitization period of 6 weeks between sequential tests recommended by World Organization for Animal Health (WOAH)^1^. The intradermal tests using PPDs were conducted in each round, and the IGRA test was added during the final two rounds (6 and 7).

**Figure 1:**
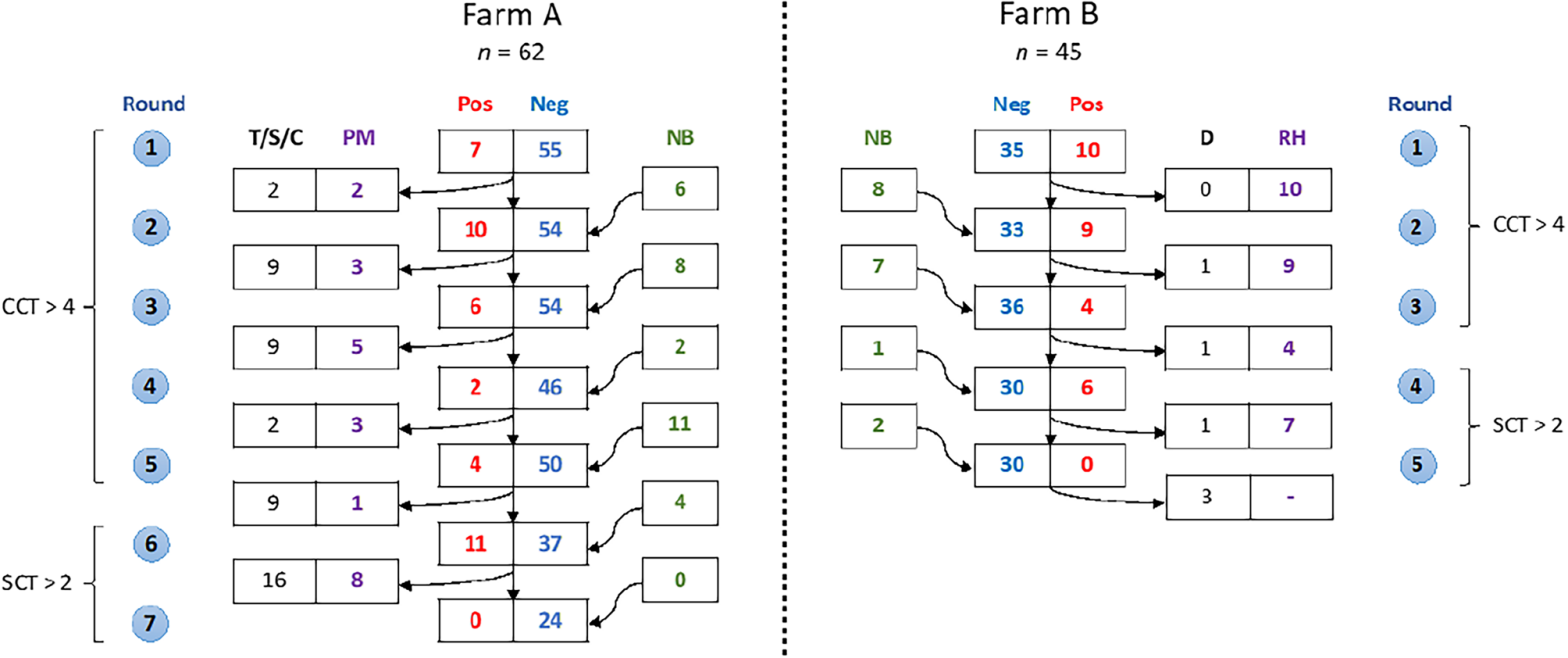
Overview of the bTB control strategies on Farm A and Farm B. TC approach was applied on Farm A, which involved seven rounds of skin testing, followed by slaughtering and postmortem examination (PM) of test positive animals. The details also include the number of newborn animals (NB) introduced during each testing round, and animals that were removed from Farm A due to non-bTB related deaths, culling, selling, or transferring (T/S/C). On Farm B, the TSg approach was implemented, involving five rounds of testing and subsequent segregation of test-positive animals into a reactor herd (RH). The figure on the right illustrates this approach, with details on the negative herd, new-born animals introduced during each round, and animals that died (D) from non-bTB causes during the control activity.

A CCT > 4 mm was used as a cut-off to remove test positive animals up to and including the 5th round in Farm A. Additionally, animals with inconclusive results (CCT: 1–4 mm) in two consecutive tests were also considered as test positive. During the 6th and 7th round final tests, the more sensitive SCT > 2 mm was used to identify additional test positive animals in the herd. Following each testing round, animals testing positive for bTB were isolated from the test negative herd and subsequently culled through slaughtering. However, there was an exception during the first round of testing on Farm A, where strict adherence to the protocol for removing test-positive animals was not followed due to the ongoing finalization process of the agreement with the farm at that time. Moreover, in an exceptional case during the 6th round of testing, one animal that tested negative on the skin test yet showed symptoms like coughing and progressive weight loss was culled from the farm on suspicion of bTB. The number of animals tested during each round, newborn calves, dead, sold and slaughtered are presented in Fig. 1 (and supplementary tables 1 and 2).

Newborn calves from bTB reactor animals were immediately separated from the dam and fed with colostrum previously collected from known bTB test negative animals, and subsequently fed with boiled milk. All newborn calves were kept separate until the next bTB screening test and were introduced into the bTB test negative herd only if they tested negative. Test negative and positive groups were attended by separate animal care takers.

A TSg approach was followed to control bTB on Farm B which was tested for a total of five rounds. Starting from the first round bTB test positive and negative animals were kept and managed separately and strict farm biosecurity measures were practiced with no introduction of purchased animals from outside. Dedicated animal attendants were assigned to oversee the test-negative and test-positive groups.

In each testing round, intradermal tests were administered, and additionally, the IGRA was incorporated during the last two rounds (4th and 5th). Screening during the first three rounds was done using CCT at cut-off > 4 mm and animals with inconclusive results (CCT: 1–4 mm) in two consecutive tests were also considered as test positive. Then in the 4th and 5th rounds, the SCT with a cut-off > 2 mm was employed to identify and segregate test-positive animals from the herd. As an exception during the 4th round of testing, one animal that tested negative on the skin test but showed clinical signs of coughing and progressive weight loss, and tested positive on the IGRA, was segregated from the negative herd, being suspected of having bTB.

Newborn calves were introduced to the test negative herd after testing confirmed their bTB negative status confirmation and were fed boiled milk. Figure 1 and supplementary table 2 provides details on the number of animals tested in each round, including those from the negative and positive herds, newly introduced calves, and deceased animals. The animals remaining in the positive herd were kept under surveillance as well and tested at all rounds.

### Tuberculin skin tests

The intradermal test was performed using avian 2500 IU/dose (PPDA) and bovine purified protein derivative at 3000 IU/dose (PPDB), purchased from Prionics/ThermoFisher Scientific, Lelystad B.V., The Netherlands (lot: 015,012 and 935,411). In large animals both antigens were injected in one side of the neck region. Whereas for calves and young animals without adequate space on one side of the neck; one antigen was injected on one side and the other antigen on the opposite side of the neck. A distance of at least 12.5 cm was maintained between two antigen injection sites. The McLintock syringe was used to inject 0.1 ml volume of each antigen, and skin thickness before and 72 h after injection was measured by same operator using each time the same manual Irish caliper.

The comparative cervical test (CCT) was considered positive when CCT > 4 mm and animals with inconclusive results (CCT: 1–4 mm) in two consecutive tests were also considered to be test positive. The results were also analysed using the more sensitive interpretation CCT > 0. As part of the CCT test interpretation a minimum PPDB reaction ≥ 2 mm was required for an animal to be bTB test positive^1^. For the single cervical test (SCT), the sensitive interpretation SCT > 2 mm was used as a cut-off.

### Interferon gamma release assay (IGRA)

For the IGRA test, whole blood samples were collected prior to the skin test using a heparinized vacutainer tube (Lithium heparin). Blood samples were transported to a laboratory within 6 h at room temperature for stimulation. The whole blood sample was stimulated with PPDB (at final concentration of 300 IU/ml) and PPDA (at a final concentration of 250 IU/ml).

In addition, each blood sample was also stimulated using pokeweed mitogen (PWM, Sigma-Aldrich) at 10 µg/ml final concentration as a positive control for stimulation and RPMI1640 media with L-Glutamine was used as negative control for the stimulation. The blood samples (250 µl) were stimulated in duplicate and the plasma supernatant was harvested after incubation.

Using the *Mycobacterium bovis* Gamma Interferon test kit for cattle BOVIGAM (Product number: 63326, Prionics, Switzerland) the amount of secreted gamma interferon in the plasma supernatant following the antigen stimulation^13^ was assayed. The test was conducted according to the manufacturer’s instructions and the absorbance was measured at 450 nm using ELISA reader. Samples were tested in duplicate and the mean optical density (OD) value was used for result analysis. The sample was considered positive if the mean OD value of PPDB-PPDA was equal to or greater than 0.1.

### Postmortem examination

Postmortem examination was carried out on the slaughtered bTB positive animals. A detailed assessment for the presence of tuberculous lesions (caseous, calcified, or abscess types) was carried out in various lymph nodes and organs. The information about the type, number and size of lesions was recorded during the postmortem examination and the severity of each lesion was scored using the semi quantitative procedure^14^. Details of the lymph nodes and organs inspected during the postmortem examination are provided with the Supplementary Fig. 1.

### Estimation of costs incurred

The costs incurred for TC on Farm A and TSg on Farm B were analyzed by estimating the major expenses during the bTB control period. The expenses related to the skin test for bTB screening were calculated for both the TC and TSg strategies, taking into account the costs of PPD and other reagents, daily allowances for field experts conducting the tests, and fuel expenses for transportation during fieldwork.

Costs associated with culling of bTB reactor animals included replacement values for the culled cattle and were estimated according to market prices. The salvage value of meat from these culled cattle, if deemed fit for human consumption following a detailed inspection by a veterinarian during slaughter, was deducted from the estimated replacement value of the culled cattle.

Costs associated with the management of segregated bTB-positive animals (in Farm B), including expenses for constructing a new barn to house the segregated cattle and the annual salaries for the caretakers of these animals were considered. All cost estimates were in US dollars and applied an exchange rate of 55.89 Ethiopian Birr (ETB) per US dollar, based on the rate from October 19, 2023.

### Data analysis

Descriptive statistics were employed to summarize the results of the bTB tests in tables. Scatter plots were used to illustrate the individual animals’ CCT and SCT responses. Additionally, a line graph was employed to demonstrate the reduction in bTB prevalence over time. These analyses were performed using the Prism 9 (GraphPad Prism version 9.4.1 (681)). Additionally, heat maps were also used to illustrate test results and outcomes for individual animals.

The scatter plot to illustrate the correlation of skin test result and total lesion score for the culled animals were performed with ggplot2 package using the R Statistical software version 4.2.2. The scatter plot that illustrates the reduction in skin test reactivity following repeated test was also prepared using ggplot2 packages. A statistical analysis with linear mixed-effects models was employed to account for repeated measures on the same subjects, with ‘Round’ as a fixed effect and a random intercept for each animal to address within-subject correlation. Post-hoc pairwise comparisons were performed using Tukey’s Honest Significant Difference method to control for the family-wise error rate due to multiple testing, and the results adjusted using the Kenward-Roger degrees-of-freedom method. *P* < 0.05 was considered for statistical significance. A rough estimation of the costs for the TC and TSg approaches was carried out by considering major expenses and summarized in table.

### Ethics statement

Ethical clearance was obtained from AAU, Akilu Lemma Institute of Pathobiology, Institutional Review Board (ALIPB IRB/76/2014/22). In addition, the Animal Research Scientific and Ethics Review Committee (ARSERC) of Animal Health Institute also reviewed the research protocol and provided ethical clearance (Ref. number: ARSERC/EC/003/26/09/2019). All the field works were conducted following international guidelines. Furthermore, the study was conducted and reported in accordance with ARRIVE guidelines.

## Results

### Both TC and TSg are effective at reducing bTB prevalence within herds when employing progressively more sensitive diagnostic thresholds

**Figure 2:**
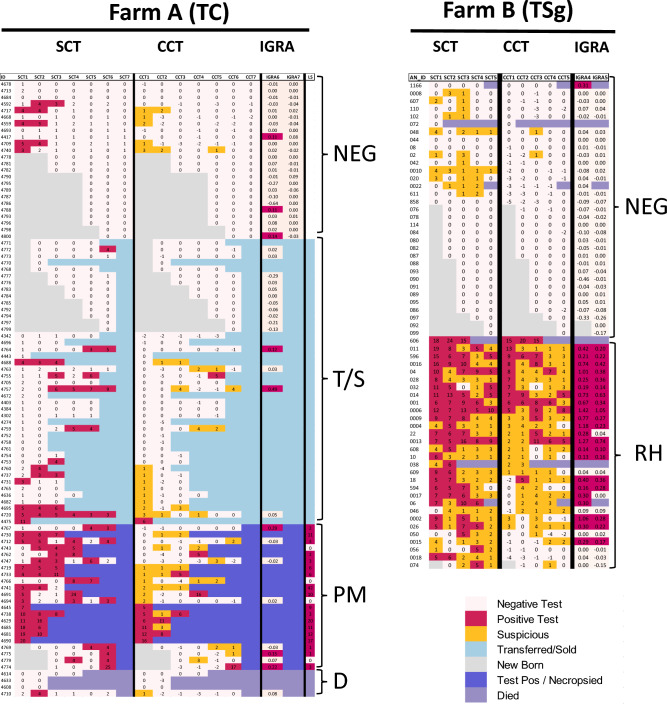
A heat map presents the test results with measures implemented as part of the bTB control strategies on both farms. Individual animal test results on both farms are shown, with positive results highlighted in red–purple, inconclusive results in yellow, and negative results in white. In Farm A, with the TC approach, the heat map shows testing outcomes, identifying animals that tested negative (NEG) and those that tested positive. The test positive animals were slaughtered and subjected to postmortem examinations (PM). Additionally, it indicates other animals, including those transferred, culled, or sold (T/S), as well as those that died (D) during the control period. The results of the skin test, IGRA, and lesion score (LS) were presented for Farm A. On Farm B, employing the TSg approach, animals are divided into test-negative (NEG) and reactor herd (RH) categories. Animals that died due to non-bTB related causes were also indicated. The heat map also indicates newborn animals in both herds. Skin test and IGRA results are shown for Farm B.

**Figure 3:**
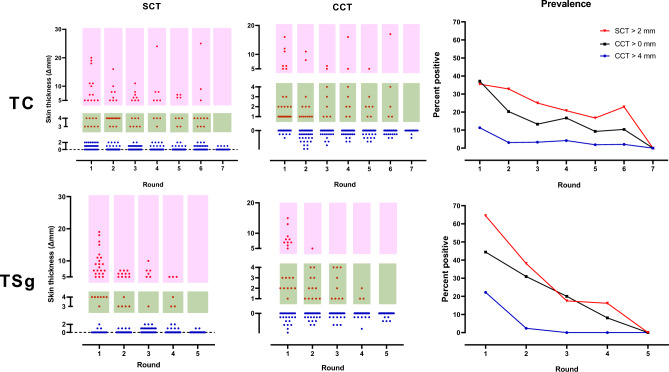
SCT and CCT responses of animals, and bTB prevalence at Farm A and Farm B. Light red data points represent animals that tested positive, and blue data points represent those that tested negative for CCT and SCT responses. Deep red data points also indicate positive animals in the SCT plots (SCT > 2 mm) and CCT plots (as per CCT > 4 mm), yet considered as inconclusive with a CCT > 4 mm cut-off. The prevalence plots show a notable decrease in bTB prevalence across all three skin test thresholds from the initial to the final testing round.

At both Farm A and Farm B, there was a significant reduction in bTB prevalence when coupled with the either culling (TC) or segregation (TSg) of reactor animals, respectively (Figs. 2, 3). On Farm A, employing the TC strategy and a CCT > 4 mm threshold, bTB prevalence decreased from 11.3% in the initial round to 0% by the seventh final round (Table 1; Fig. 3). While the number of reactor animals identified using this threshold showed a rapid decline from the second round of testing, at least one positive animal was detected until the sixth round. In contrast, on Farm B’s negative herd, the TSg strategy led to a noticeable reduction in bTB prevalence, from 22.2% in the first round to 2.4% in the second round, reaching and maintaining 0% from the third round through to the final, fifth round, using the same CCT > 4 mm threshold (Table 1; Fig. 3).

**Table 1:** Summary of bTB skin test results at Farm A and Farm B.

Farm A (TC)							
Test round	Round 1	Round 2	Round 3	Round 4	Round 5	Round 6	Round 7
Number of animals	62	64	60	48	54	48	24
Test	Number of test positives (%)						
SCT > 2 mm	22 (35.5)	21 (32.8)	15 (25)	10 (20.8)	9 (16.7)	11 (22.9)	0 (0)
CCT > 0 mm, ΔB ≥ 2	23 (37.1)	13 (20.3)	8 (13.3)	8 (16.7)	5 (9.3)	5 (10.4)	0 (0)
CCT > 4 mm	7 (11.3)	2 (3.1)	2 (3.3)	2 (4.2)	1 (1.9)	1 (2.1)	0 (0)
IGRA	–	–	–	–	–	8 (16.6)	0 (0)

Farm B (TSg)							
Test round	Round 1	Round 2	Round 3	Round 4	Round 5		
Number of animals	45	42	40	37	30		
Test	Number of test positives (%)						
SCT > 2 mm	29 (64.4)	16 (38.1)	7 (17.5)	6 (16.2)	0 (0)		
CCT > 0 mm, ΔB ≥ 2	20 (44.4)	13 (31.0)	8 (20)	3 (8.1)	0 (0)		
CCT > 4 mm	10 (22.2)	1 (2.4)	0 (0)	0 (0)	0 (0)		
IGRA	–	–	–	4 (10.8)	0 (0)		

While both farms achieved a test-negative status using a CCT > 4 mm threshold, the employment of more sensitive thresholds, such as CCT > 0 mm and SCT > 2 mm, revealed the presence of additional test-positive animals. For example, even after several rounds of testing and culling at Farm A based on the CCT > 4 mm threshold, 22.9% and 10.4% of animals remained test positive when SCT > 2 mm and CCT > 0 mm thresholds were applied in the sixth round, respectively. Additionally, 16.6% tested positive using the standard PPD IGRA in the same round. The adoption of the SCT > 2 mm threshold in the sixth round was instrumental in identifying the residual test positive animals, leading to a test-negative status across all tests by the seventh round (Table 1; Fig. 3).

Similarly, at Farm B, despite all animals testing negative from the third round onwards using the CCT > 4 mm threshold, more sensitive testing revealed undetected test-positive animals within the herd. Specifically, in the third round, 17.5% and 20% of animals tested positive using SCT > 2 mm and CCT > 0 mm thresholds, respectively. The presence of these test-positive animals in the herd, identified as negative by less sensitive diagnostic thresholds, posed a significant risk for bTB transmission to susceptible animals.

It was not until the fifth and final round of testing that Farm B achieved a test-negative status across all testing criteria. This was primarily attributed to the shift in screening criteria from CCT > 4 mm to SCT > 2 mm threshold in the fourth round (Table 1; Fig. 3). This underscores the importance of using more sensitive diagnostic thresholds to ensure comprehensive detection and management of bTB infections.

The postmortem findings from animals culled on Farm A underscores the importance of selecting appropriate cut-off values for skin testing with tuberculin. Over 18 months, 69 animals were removed, 26 of which because of positive bTB tests, while the rest were sold, died of other causes, or were removed for non bTB reasons (Figs. 1, 2; Supplementary Table 1). Of the 26 (22 female and 4 male) bTB reactor cattle, postmortem examination and lesion scoring were performed on 22, of which 18 showed visible lesions and 4 did not (Supplementary Fig. 1). Skin test results showed that only 40.9% of the slaughtered animals were test positive with CCT > 4 mm, whereas a higher detection rate was observed with the more sensitive (severe) test interpretations of CCT > 0 (81.2%) and SCT ≥ 2 mm (91%).

**Figure 4:**
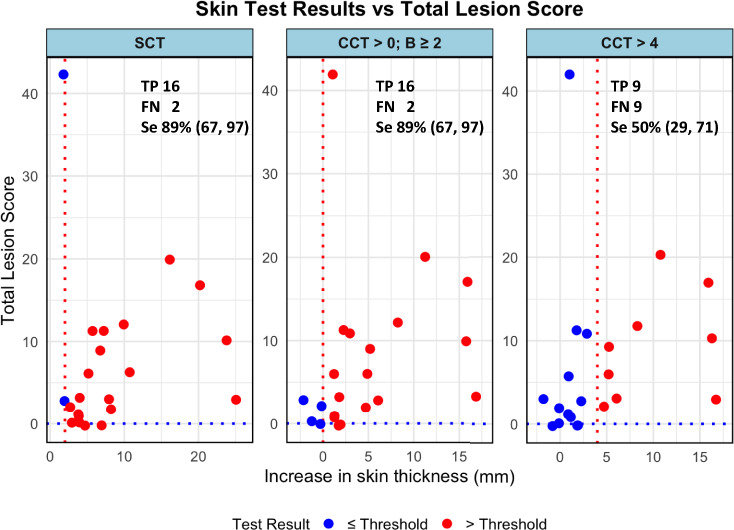
Correlation between diagnostic test results and total lesion score in slaughtered animals on Farm A. This scatter plot displays the relationship between the increase in skin thickness as measured by different intradermal tests and the total lesion score observed during postmortem examinations of 18 cattle with visible bTB lesions. Blue dots represent animals with test results below the threshold, while red dots indicate results above the threshold, deemed true positives (TP) for bTB. The single cervical test (SCT) with a > 2 mm cut-off and the comparative cervical test (CCT) with a > 0 mm cut-off successfully identified 88.9% of the animals with lesions, marked as TP (16 out of 18), demonstrating high sensitivity (Se) of 89% with a 95% confidence interval (CI) of (67, 97). In contrast, the CCT with a > 4 mm cut-off detected only 50% of lesioned animals as TP (9 out of 18), indicating a lower sensitivity of 50% (CI: 29, 71). False negatives (FN) are also noted for each test, with 2 FN for SCT, 2 FN for CCT > 0, and 9 FN for CCT > 4 mm, which are cases where the test failed to identify the disease despite the presence of lesions. The red dashed vertical lines mark the thresholds for the respective tests, while the blue dashed horizontal lines distinguish between animals with positive and negative lesions.

Furthermore, among the 18 animals that showed visible lesions during postmortem examination, the highest percentage (88.9%) of lesioned animals was detected by both SCT > 2 mm and CCT > 0. In contrast, the CCT > 4 mm detected only 50% of these cases (Fig. 4; Supplementary Table 3).

### A substantial reduction in skin test reactivity due to repeated testing of bTB positive cattle is observed

**Figure 5:**
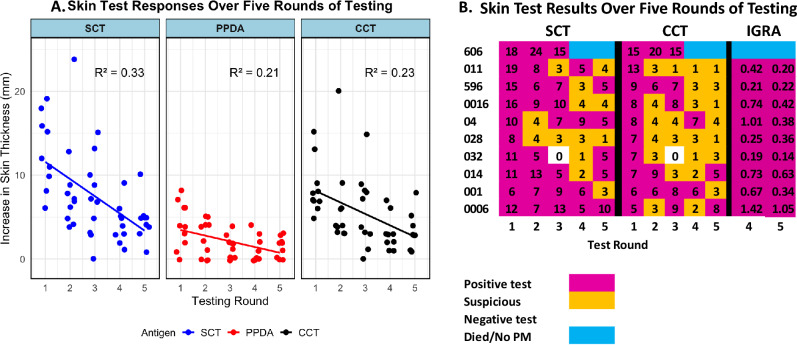
Temporal desensitization of tuberculin skin test reactivity in bTb-positive cattle. (**A**) The plot displays changes in skin test reactivity measured by the increase in skin thickness (in millimeters) over five consecutive testing rounds for SCT (blue circles), PPDA (red circles), and CCT (black circles). Each point represents the reaction of an individual animal at each round of testing. The regression lines indicate the trend over time, with the corresponding R-squared values denoting the proportion of variance explained by the model for each antigen. Statistical analysis via linear mixed-effects models reveals the presence of desensitization, with significant differences in skin test responses between the first round and subsequent rounds (refer to Supplemental Table 4 for detailed statistical results). (**B**) The heat map demonstrates gradual decrease in both SCT and CCT results across five rounds of repeated skin testing conducted on the initial group of 10 bTB positive animals. However, all animals were test positive in the IGRA test, which was conducted only during the fourth and fifth round tests. For the IGRA test, an OD value of 0.1 or greater is considered positive.

An important component of any control strategy, particularly the TSg approach, is the need for a continued monitoring of suspect and reactor animals with the herd. In Farm B’s positive herd, there were 10 bTB positive animals (CCT > 4 mm) already segregated in the first round that were re-tested during an additional four rounds, providing an opportunity to explore the temporal changes in skin test responses to repeated testing with bovine and avian tuberculins. Desensitization responses with the SCT, CCT and PPD-A were observed over five rounds of testing for these ten individuals (Fig. 5).

The linear mixed-effect model and mean difference analysis using ANOVA shows a statistically significant decrease in the skin test response over time for each antigen, indicative of a desensitization effect (supplementary tables 4 and 5). For SCT, significant decreases were observed from Round 1 to Rounds 3, 4, and 5. CCT showed significant decreases later, starting from Round 4, while PPDA exhibited a significant decrease from Round 3 onwards. These findings suggest a trend toward desensitization across all antigens, with the initial response significantly diminishing by rounds 4 and 5.

Despite the observed decrease in skin test reactivity among the ten bTB-positive cattle, concurrent testing with IGRA during the fourth and fifth rounds provided an additional diagnostic perspective. Although the skin test responses diminished progressively following repeated tuberculin skin testing, all nine animals consistently tested positive with the standard PPD IGRA in the last two rounds. Remarkably, even with the reduced skin thickness reactions, eight out of the nine reactors (approximately 89%) were still identifiable using the criteria of CCT greater than 0 mm and SCT greater than 2 mm in the fifth round. In contrast, only two reactors of the nine remaining reactors were detectable when applying the less stringent CCT cutoff of greater than 4 mm. These results underscore the potential for desensitization effects in tuberculin skin tests and highlight the importance of complementary diagnostic approaches like IGRA in the reliable detection of bTB, particularly during serial testing scenarios.

### TC and TSg both require substantial upfront costs, with TC costs exceeding those of TSg

A comparison of cost estimates between test-and-cull (TC) and test-and-segregation (TSg) strategies reveals a stark contrast in major expense categories incurred to reach a test-negative bTB status (Table 2). The TC strategy incurred substantial costs in testing, with a total of $3,799, but notably high costs associated with culling of $34,720, driven primarily by the replacement value of culled cows and bulls, and offset only slightly by the salvage value of meat. In total, the TC costs amounted to $38,520, with ~ 90.1% of the total costs attributed to culling. In comparison, the total costs associated with the TSg strategy on Farm B to reach test-negative status was $14,045 that included testing costs of $2,737 and avoided culling associated costs entirely. Instead, major expenses with the TSg approach were related to barn construction and animal attendant salaries, totaling $11,308 and constituting ~ 80.5% of the total TSg expenses that were not incurred with the TC approach (Table 2).

**Table 2:** Comparative cost analysis of TC versus TSg strategies.

Category	Item description	Unit Cost	TC (n)	TC ($)	TSg (n)	TSg ($)
Skin testing	Reagent, PPD (per test)	1.79	360	644	270	483
	Other consumables	4.47	7	31	5	22
	Per-diem (4 persons × 7d /round)	46.52	49	2279	35	1,628
	Fuel (450 km each round), 90L/ round	1.34	630	845	450	603
	Subtotal for testing			$ 3799		$ 2737
Culling	Replacement value of culled cows	1,520.84	22	33,459		–
	Replacement value of culled bulls	715.69	4	2863		–
	Less salvage value of meat (price of kg/live weight × 360 kg per animal)	64.04	25	1601		–
	Subtotal for culling			$34,720		–
Segregation	Barn construction	8946		–	1	8946
	Annual salary for animal attendant ($98.41 per month)	1,180.92		–	2	2362
	Subtotal for segregation			–		$11,308
Total costs to reach herd test-negative status				$38,520		$14,045
Per capita costs (Total/animals tested to reach test-neg status)			360	$ 107	270	$ 52

A breakdown of costs associated with the implementation of TC and TSg strategies for bTB control over an 18-month period at Farm A and 12-month period in Farm B are presented. Per capita costs were calculated by dividing the total expenses by the number of animals tested to achieve this status to compare herds of different sizes and testing rounds and were based on 360 animal testing over 18-months for TC while TSg estimates were derived from 270 animals over 12-months.

Since the numbers of animals in each of the herds and the numbers of rounds of testing were different, for ease of comparability, total costs were divided by the number of animals tested across all rounds, resulting in estimated per capita costs of $107 for TC and $52 for TSg. While not generalizable since these were not representative herds, this “normalized” costing approach suggests a two-fold better economic efficiency of TSg in Farm B, highlighting it as a more cost-effective strategy for achieving a test-negative bTB status when compared to TC in Farm A when suitable land and labor is available for the establishment of separate test-negative and reactor herds. In situations where barn facilities exist and labor is available to ensure proper segregation of negative from reactor animals, the upfront costs for implementing this approach may be further substantially lowered.

## Discussion

Ethiopia is well known for its large cattle population estimated to be around 66 million cattle, of which ~ 2 million are exotic or cross breed cattle^15^. According to a systematic review and meta-analysis^16^ the estimated prevalence of bTB in the country was 5.8% (95% CI 4.5, 7.5), suggesting that approximately 3.8 million cattle may be infected with bTB in Ethiopia. However, a higher bTB prevalence of 21.6% (95% CI 14.7–30.7) was estimated for herds with high milk producing exotic breed Holstein–Friesian and cross breed cattle, routinely kept under an intensive management system. Another recent study reported a similar higher bTB prevalence of 24.5% (95% CI 23.3–25.8) in intensive dairy farms located in Central Ethiopia^17^. All these prevalence estimates rely on the use of the CCT using a > 4 mm cut-off, and applying more sensitive interpretations of the intradermal assays such as a CCT > 0 mm or an SCT > 2 mm are likely to show the true prevalence to be higher than current estimates. Regardless, the high bTB prevalence figures in the specialized dairy herd underscore the urgency for the implementation of a comprehensive nationwide bTB control strategy in Ethiopia.

This study was undertaken to explore options for bTB control approaches/interventions that could be introduced in Ethiopia and other similarly situated LMICs. To this purpose, both the test-and-cull and the test-and-segregation approaches were implemented on two dairy farms to reduce the prevalence of bTB.

Utilizing either the TC or the TSg approach resulted in a significant reduction in the prevalence of bTB on the respective farms. However, applying progressively more sensitive diagnostic cut-offs for the intradermal skin tests was essential to achieve this result. For example, when applying a cut-off of CCT > 4 mm Farm B was already test negative after the 3rd round of testing, but still 17.5% and 20% of the animals were test positive in the same round when applying SCT > 2 mm and CCT > 0 mm thresholds, respectively. This suggests that test positive animals most likely bTB-infected but not detected by using a cut-off of CCT > 4 mm, were still present in the negative herd. Similar findings were also observed on Farm A, and, again, it was only after the introduction of the more sensitive threshold of SCT > 2 mm that the remaining test positive animals could be removed. However, it is important to recognize that even while no reactors were detected in either herd under the more sensitive cut-offs, confirming the true bTB-free status of herd will necessitate additional testing. For instance, as per EU regulations, all animals within the herd must test negative on two intradermal tests spaced 60 days apart and annual retesting of the whole herd is required over a prolonged period in countries that have not reached an Officially Tuberculosis Free (OTF) status^18^. Similar guidelines are not currently available for certification of herds in Ethiopia and need to be developed as part of a national control program.

In retrospect, the use of sensitive intradermal skin test like CCT > 0 (with PPD B ≥ 2 mm) or SCT > 2 mm at the start of bTB control activity would have been helpful to detect most, if not all, of bTB positive animals in both high prevalence dairy farms. However, given the concern at the start of the project about the true bTB status of both herds and whether a strict application of the skin test, in combination with the imperfect specificity and sensitivity of the available diagnostic tools, resulting in the culling of false-positive animals or missing truly infected ones, would have resulted in an unacceptably large reduction in herd size (~ 40% of the animals in both herds were reactors when considering the more sensitive cut-offs), and considered as untenable.

Postmortem examination revealed lesions in 82% (18/22) of the culled reactors at Farm A. In contrast, lesions were not observed in 4 of 22 (18%) of culled reactors animals, a finding that may be attributed to early infection, overlooked smaller size lesions during gross pathological examination, cross reaction with environmental *Mycobacterium* species, etc.^19,20^.

The ante mortem test result of the 22 slaughtered animals just prior to slaughter showed that while only ~ 41% were test positive by CCT > 4 mm, most were identified as reactors by the more sensitive interpretations such as the SCT > 2 mm (91%) and the CCT > 0 (81.2%). A high percentage (88.9%) of visibly lesioned animals was similarly detected by using the SCT > 2 mm and the CCT > 0 mm. This illustrates the importance of using more sensitive diagnostic thresholds for the detection of bTB infected and lesioned (and likely infectious) animals which would otherwise remain in the herd and continue to spread infection.

Since Farm A is a herd with no prior interventions, the animals were likely infected for longer periods. Consequently, the PPDb responses are high, and a large proportion of the reactors showed extensive visible lesions at necropsy. As shown in Fig. 4, most of those animals were detected by applying SCT > 2 mm and CCT > 0 mm, and would also have been detected as lesioned bTB-infected animals at slaughter. Hence, the fraction of lesioned animals in Farm A is very high when compared to slaughterhouse surveillance results from European countries with yearly testing intervals followed by culling of test-positive animals for bTB^21,22^.

Prior studies, including^23^, have documented desensitization to the tuberculin skin test in naturally infected cattle, after repeated skin testing at short intervals indicating the need to maintain an interval of at least 8 weeks or 60 days in between testing, as required by the WOAH guidelines^1^, to maintain an optimal sensitivity of the skin test essential for bTB control programs. After repeated short-interval testing (range 2–4 months) at Farm B on bTB-positive animals showed a consistent and significant decrease in skin thickness reactions to bovine and avian tuberculin. This diminution in reaction magnitude underscores the need for sufficiently long intervals between consecutive skin tests to avert skin desensitization. Despite the observed gradual decrease in skin induration, the more sensitive diagnostic approaches such as CCT > 0 mm, SCT > 2 mm, and tuberculin based IGRA were able to effectively identify positive animals across multiple test iterations. Specifically, animals exhibiting smaller initial reactions may be recorded as false negatives if subjected to subsequent frequent short-interval testing. Extending the period between test sessions to at least every 6 months could help mitigate reductions in skin test responsiveness, enhancing bTB management efforts, but this needs to be empirically tested in situations such as exist in Ethiopian dairy farms. Additionally, our results suggest that employing IGRA alongside or in rotation with intradermal tests may offer further benefits in detecting infected animals when there is a need for repeated testing.

In addition to desensitization, it has been reported that some animals in an advanced stage of the disease can enter a condition known as “anergy”, where they fail to react to the tuberculin despite extensive disease^19,24,25^. However, true anergy would imply a lack of response to mitogens such as pokeweed mitogen (PWM) in addition to specific antigens. In this study, the animals responded to both non-specific and specific antigens in the IGRA, indicating that true anergy was not the case. During the third round of testing, the ten initially skin test-positive animals from Farm B’s positive herd were also tested using the IDEXX ELISA. The findings show that 4 out of 10 (40%) animals tested positive before the skin test, increasing to 70% (7 out of 10) two weeks after the skin test (results not shown). The anamnestic rise in antibody levels due to a prior tuberculin skin test increases the sensitivity of ELISA tests and has been documented in previous studies^26–28^. Hence, while true anergy was not observed, serological tests are beneficial as they complement skin tests by identifying animals with low or fluctuating immune responses, particularly in chronically infected herds. This approach helps detect additional infected animals that might otherwise be missed by skin tests alone, contributing to more effective bTB control.

The cost analysis revealed that the Test and Cull strategy implemented on Farm A incurred costs twice as high as the Test and Segregate approach used on Farm B. The predominant expense in the TC strategy stemmed from the culling of test-positive dairy cattle. However, in Ethiopia’s current context, it is impractical to slaughter high-yielding, test-positive dairy cows that show no clinical signs of bTB, as their milk is typically pasteurized for human consumption. Moreover, valuable test-positive cows without clinical signs of bTB can still birth calves, which are then immediately separated and raised on milk replacers to reduce risk of horizontal transmission. Additionally, there are potential risks with significant costs, such as the possibility of replacing culled cows and bulls with animals of unknown bTB history and poor genetic performance if artificial insemination is not used by the farm under the TC approach. Furthermore, a holistic assessment of bTB control strategies must consider factors beyond direct costs. These include the welfare and health of the animals, the economic and pragmatic sustainability of the control method, the potential for increased disease transmission during culling or segregation, and ethical and societal conditions. The socioeconomic conditions in Ethiopia and other LMICs often preclude the slaughter of cattle and further complicate the implementation of a TC strategy. Hence, while our study focused primarily on the feasibility and direct costs of implementing the TC versus TSg approaches in high-burden intensive dairy production herds in Ethiopia, it is important to recognize these additional factors together with the regulatory framework needed for successful adoption of these approaches as part of an official control strategy.

Given the high bTB prevalence reported in several private dairy farms in Central Ethiopia^17,29,30^, a TC approach could lead to the culling of a substantial portion of a farm’s livestock, including its most productive cows. Therefore, implementing TC nationwide as a control strategy for bTB in Ethiopia is considered unfeasible due to its economic impact and the potential loss of valuable dairy stock.

Nevertheless, private dairy farms in the central part of Ethiopia, characterized by a high prevalence of bTB, seek affordable approaches for bTB control to mitigate associated losses in milk production, body condition, and mortality^30–32^. Furthermore, sale of animals in poor body condition often results in rejections of carcasses during meat inspections at abattoirs. Moreover, the frequent occurrence of animal deaths prompts livestock insurance companies to discontinue policies due to the excessive compensation costs, and the zoonotic risk posed by bTB further compounds the urgency for effective control measures.

Consequently, there is strong interest among private dairy farm owners to employ bTB control programs that are economically feasible. The potential for creation of market-based incentives, including through certification for bTB-free herds for premium milk and bTB-free heifers, presents a potential strategy to enhance engagement in bTB control initiatives and provide new market access.

As was also observed in Farm B, the TSg approach enables retention and utilization of bTB test-positive yet productive animals, provided they exhibit no clinical signs of bTB^7^. This approach permits dairy farms, even those with a high prevalence of bTB, to sustain economic viability by leveraging the productivity of bTB-positive animals. Given the difficulty in acquiring bTB-negative heifers of improved breeds like Holstein–Friesian for replacement stock in Ethiopia, farmers find it more advantageous to breed from productive bTB-positive cows. By immediately segregating newborn calves and feeding them with pasteurized or boiled milk alongside high-quality feed, farms can cultivate their own bTB-negative replacement stock^7,33^. On Farm B, this methodology resulted in approximately 50% of the negative herd, during the final testing round, comprising young calves that had been segregated at birth from their positive dams and nourished on boiled milk. Thus, it is vital to carefully manage the production and maintenance of disease-free young stock, especially in high bTB prevalence settings.

However, it is important to note that segregated bTB-positive animals retained on the farm alongside the bTB-negative herd pose a potential risk for reinfection of animals in the negative herd, particularly if strict biosecurity measures are inadvertently compromised. Additionally, there could be zoonotic risks for workers. Consequently, it is imperative to not only monitor and cull animals displaying potential clinical signs, but also to methodically decrease the population of test-positive animals on the farm while concurrently expanding the cohort of disease-free livestock. While this will be likely context dependent, further research is needed to ascertain the optimal duration for retaining infected animals within the herd under various scenarios, thereby ensuring the development of best-practices for effective bTB management and control using TSg and TC combination approaches.

### Study limitations

While our study provides key foundational information regarding the feasibility and effectiveness of alternative approaches to control bTB in resource-limited settings, there are several important limitations. First, the study’s limited scope, focusing on just two government-owned farms, constrains the generalizability of its conclusions across Ethiopia’s diverse dairy farming landscape. This limitation is significant, given the operational and economic disparities between government-run and privately owned intensively managed dairy farms. The latter are driven primarily by profit, presenting unique challenges such as animal movement management, new animal integration, and the implementation of stringent biosecurity measures to curb disease transmission. These challenges are especially pronounced due to the spatial and operational constraints inherent to private dairy farming in the rapidly expanding milk shed regions of LMICs.

The study’s duration is also a major limitation, with the 18-month and 12-month periods for TC and TSg strategies, respectively, being insufficient to grasp the long-term effectiveness and economic ramifications of these approaches. The dynamic nature of bTB, influenced by various factors including herd management and environmental conditions, underscores the necessity for longer-term research to accurately assess the sustainability and effectiveness of bTB control strategies. These should not only evaluate TC and TSg but also explore alternative strategies including vaccination and chemoprophylactic approaches and incorporating dynamic prediction models to better understand the potential efficacy, challenges, and economic implications of various interventions. Recent studies have reported promising findings on the use of BCG vaccination as a potential bTB control strategy. Vaccination of calves has been shown to reduce the severity of bTB lesions under natural transmission studies^34^. Our recent natural transmission experimental studies have demonstrated that BCG vaccination decreases bTB transmission, improving the prospects of elimination^35^. These findings, along with encouraging results related to a defined antigen skin test that can differentiate between infected and vaccinated animals^36,37^, suggest the need for the implementation of integrated control programs that include TSg with BCG vaccination schemes in LMICs such as Ethiopia.

It is noteworthy that evidence illustrating the potential for eliminating bTB from herds using a combination of chemotherapy and chemoprophylaxis in high value herds or certain socioeconomic settings^38^, this approach has long been considered impractical to implement due to potential costs and concerns regarding spread of drug resistance. However, with the growing drive for intensification and rapidly increasing bTB prevalence in regions where TC is unfeasible, there may be an opportunity to explore these again with anti-mycobacterial agents that are not used in human medicine.

Another critical limitation is approach to determining optimal diagnostic thresholds with the sole focus to reduce skin test reactor prevalence. While clearly establishing the importance of adopting more sensitive thresholds for identifying bTB reactors, the study was not designed to rigorously assess the potential for increased false positives and the subsequent economic and animal welfare implications, a gap that is particularly relevant given the need for a balanced understanding of diagnostic test sensitivity and specificity in regions where livestock provide a myriad of economic and developmental benefits, and the availability of replacement animals from disease free herds is particularly challenging.

Finally, while the study provides a rough cost analysis, it was not designed to consider all relevant economic or societal factors, including long-term cost savings from reduced bTB prevalence, the potential impact on animal productivity, or the broader economic benefits of improved animal and public health. This indicates a pressing need for a comprehensive economic analysis to offer a more detailed understanding of the cost-effectiveness of various alternative approaches to bTB control.

### Concluding comments

Overall, the most effective control regime always depends upon epidemiological and economic circumstances. Collectively, our findings underscore a need for further research on alternative strategies for bTB control that are tailored to the specific conditions of diverse dairy farming operations in Ethiopia and other similarly situated LMICs that do not currently have established bTB control programs. Future studies should aim to thoroughly evaluate the feasibility, economic implications, and practicality of implementing TSg and other alternative bTB control strategies, including conducting detailed cost–benefit analyses that consider the operational and economic dimensions of various dairy farming practices. This knowledge is essential for developing a comprehensive understanding and evidence base for how different bTB control strategies may contribute to sustainable livestock and zoonotic disease management, thereby supporting broader social and economic development goals.

### Supplementary Information

Supplementary Information.

## Data Availability

All the analyzed data have been incorporated into the manuscript and supplementary materials. For additional inquiries, the corresponding authors can be contacted.

## Abbreviations

bTB: Bovine tuberculosis
CCT: Comparative cervical test
CFT: Caudal fold test
IGRA: Interferon gamma release assay
LMICs: Low and middle income countries
MTBC: *Mycobacterium tuberculosis* complex
PPD: Purified protein derivative
SCT: Single cervical test
SIT: Single intradermal test
TC: Test-and-cull
TSg: Test-and-segregate
TST: Tuberculin skin test
WHO: World health organization
WOAH: World organisation for animal health
