# Supplementary material for: Assessing the feasibility of test-and-cull and test-and-segregation approaches for the control of high-prevalence bovine tuberculosis in Ethiopian intensive dairy farms
Source: Sci Rep. 2024 Jun 21;14:14298. doi: 10.1038/s41598-024-64884-x (PMC11192749; doi:10.1038/s41598-024-64884-x)
Supplement: Supplementary file 1 — Supplementary Information. [file 41598_2024_64884_MOESM1_ESM.docx]

**SUPPLEMENTARY TABLES AND FIGURES**

**Tables S1-S5**

**Fig S1**

Assessing the feasibility of test-and-cull and test-and-segregation approaches for the control of high-prevalence bovine tuberculosis in Ethiopian intensive dairy farms

Matios Lakew^1,2*^, Biniam Tadesse^2^, Sreenidhi Srinivasan^3^, Muluken Aschalew^4^, Bekele Andarge^4^, Dirshaye Kebede^5^, Addisu Etifu^5^, Tena Alemu^5^, Bekele Yalew^2^, Teferi Benti^2^, Abebe Olani^2^, Shubisa Abera^2^, Wegene Bedada^2^, Abebe Fromsa^1^, Getnet Abie Mekonnen^2^, Gizat Almaw^2^, Gobena Ameni^1,6^, Hagos Ashenafi^1^, Balako Gumi^1^, Douwe Bakker^7,8*^ and Vivek Kapur^9.10*^

^1^Aklilu Lemma Institute of Pathobiology, Addis Ababa University, P.O. Box 1176, Addis Ababa, Ethiopia. ^2^Animal Health Institute, P.O. Box 04, Sebeta, Ethiopia. ^3^Global Health Initiative, Henry Ford Health, Detroit, MI 48202, USA. ^4^Livestock Development Institute, Bishoftu, Ethiopia. ^5^Wolkite University, Gubre, Ethiopia. ^6^Department of Veterinary Medicine, College of Agriculture and Veterinary Medicine, United Arab Emirates University, PO Box 15551, Al Ain, United Arab Emirates. ^7^Independent Researcher and Technical Consultant, Lelystad, Netherlands. ^8^Departamento de Sanidad Animal, Facultad de Veterinaria, Universidad Complutense, Madrid, Spain. ^9^Department of Animal Science, The Pennsylvania State University, University Park, PA, United States. ^10^Huck Institutes of the Life Sciences, The Pennsylvania State University, University Park, PA, USA.

*Correspondence: Matios Lakew: matioslakew@gmail.com, Douwe Bakker: douwe.bakker@kpnmail.nl, Vivek Kapur: vxk1@psu.edu

Supplementary Table 1: Summary of the bTB control efforts on Farm A, including detail on the number of animals in each category

| **Farm A** | **R1** | **R2** | **R3** | **R4** | **R5** | **R6** | **R7** | **Total** |
| --- | --- | --- | --- | --- | --- | --- | --- | --- |
| Test Positive | 7 | 10 | 6 | 2 | 4 | 11 | 0 | 40 |
| Test Negative | 55 | 54 | 54 | 46 | 50 | 37 | 24 |  |
| Postmortem examination on bTB positive (PM) |  | 2 | 3 | 5 | 3 | 1 | 8 | 22 |
| Non-bTB sold, transferred, or culled |  | 2 | 7 | 9 | 1 | 9 | 15 | 43 |
| Dead |  | 0 | 2 | 0 | 1 | 0 | 1 | 4 |
| New born calves |  | 6 | 8 | 2 | 11 | 4 | 0 | 31 |
| Total in Herd | 62 | 64 | 60 | 48 | 54 | 48 | 24 |  |

Supplementary Table 2: Summary of the bTB control effort on Farm B, including detail on the number of animals in each category

| **Farm B** | **R1** | **R2** | **R3** | **R4** | **R5** | **Total** |
| --- | --- | --- | --- | --- | --- | --- |
| Test Positive | 10 | 9 | 4 | 6 | 0 | 29 |
| Test Negative | 35 | 33 | 36 | 30 | 30 |  |
| Transferred to Pos herd | 10 | 9 | 4 | 7 | 0 | 30 |
| Dead from Neg herd | 0 | 1 | 0 | 0 | 2 | 3 |
| Newborn calves |  | 8 | 7 | 1 | 2 | 18 |
| Total in Neg herd |  | 42 | 40 | 37 | 30 |  |
| Total in Pos herd | 10 | 19 | 22 | 28 | 27 |  |
| Dead from Pos herd | 0 | 0 | 1 | 1 | 1 | 3 |

Supplementary Table 3: Summary of the intradermal skin test results and visibly lesioned animals

| **Test** | **No. (%) of test positives** | **% with visible lesions in test positives** | **No. (%) of lesioned animals detected by test** |
| --- | --- | --- | --- |
| SCT > 2 mm | 20/22 (91) | 80 | 16/18 (88.9) |
| CCT > 0 mm, ΔB > 2 | 18/22 (81.2) | 88.9 | 16/18 (88.9) |
| CCT > 4 mm | 9/22 (40.9) | 100 | 9/18 (50) |

Supplementary Table 4: Statistical analysis of repeated test results reveals the presence of desensitization

| **Antigen** | **Term** | **Estimate** | **Std. Error** | **Statistic** | **P-value** | **R-squared** | **Adj. R-squared** |
| --- | --- | --- | --- | --- | --- | --- | --- |
| SCT | Intercept | 13.6 | 1.42 | 9.62 | < 0.001 | 0.33 | 0.31 |
| SCT | Round | -2.05 | 0.44 | -4.71 | < 0.001 | 0.33 | 0.31 |
| PPDA | Intercept | 4.15 | 0.64 | 6.49 | < 0.001 | 0.21 | 0.19 |
| PPDA | Round | -0.68 | 0.2 | -3.47 | < 0.001 | 0.21 | 0.19 |
| CCT | Intercept | 9.47 | 1.21 | 7.8 | < 0.001 | 0.23 | 0.21 |
| CCT | Round | -1.37 | 0.37 | -3.67 | < 0.001 | 0.23 | 0.21 |

Supplementary Table 5: Comparison of mean skin test thickness differences across test rounds using one-way ANOVA.

| **Test** | **Mean skin thickness** | | | | | **Comparison rounds** | **Mean difference** | **P Value** |
| --- | --- | --- | --- | --- | --- | --- | --- | --- |
|  | **R1** | **R2** | **R3** | **R4** | **R5** |  |  |  |
| SCT | 12.6 | 8.7 | 7.2 | 4.2 | 4.7 | R1 vs. R4 | 8.378 | 0.0009 |
|  |  |  |  |  |  | R1 vs. R5 | 7.933 | 0.0018 |
| CCT | 8.5 | 6.2 | 5.8 | 3.1 | 3.2 | R1 vs. R4 | 5.389 | 0.0226 |
|  |  |  |  |  |  | R1 vs. R5 | 5.278 | 0.0267 |
| PPDA | 4.1 | 2.5 | 1.4 | 1.1 | 1.4 | R1 vs. R3 | 2.7 | 0.0093 |
|  |  |  |  |  |  | R1 vs. R4 | 2.989 | 0.0047 |
|  |  |  |  |  |  | R1 vs. R5 | 2.656 | 0.0134 |

A statistically significant reduction in mean skin induration was observed for both PPD B and PPD A antigens after short-interval repeated tests, starting from round 3 for PPD A and from round 4 for CCT and SCT, compared to the initial test round. This suggests the occurrence of desensitization following short-interval repeated skin tests.


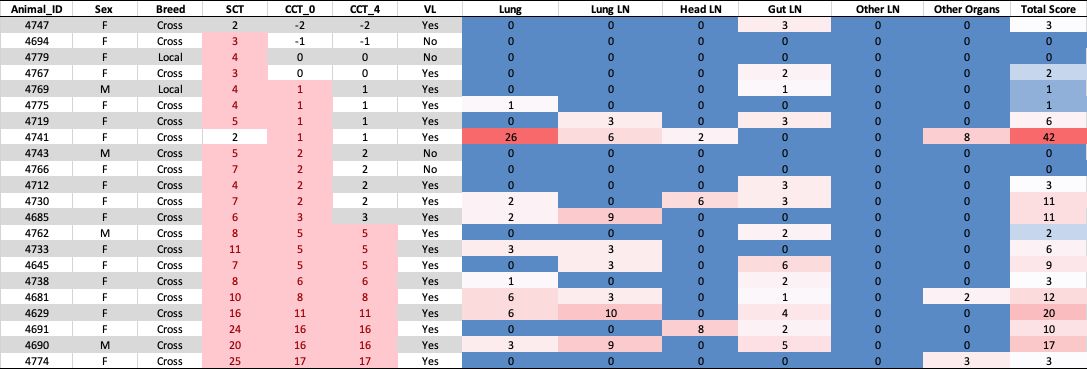


Supplementary Figure 1: Heat map showing the skin test result and lesion score of slaughtered animals on Farm A. The details of the lymph nodes and organs inspected during the postmortem examination are described below.

The lung (the cranial, cardiac, diaphragmatic and accessory lobes in the right lung and the cranial, cardiac and diaphragmatic lobes in the left lung) was examined for the presence of tuberculous lesions. Furthermore, lung associated lymph nodes, including the cranial, middle and caudal mediastinal lymph nodes, bronchial and trachea-bronchial lymph nodes, were also inspected. Head associated lymph nodes examined included: mandibular, parotid, lateral retropharyngeal and medial retropharyngeal lymph nodes in both left and right sides. Additionally, gut-associated lymph nodes, including the mesenteric and hepatic lymph nodes, were scrutinized for lesion presence. External lymph nodes like the pre-scapular, pre-femoral and deep popliteal were examined for gross pathological lesions. Visceral organs like the liver and kidneys were also inspected for the presence of tubercle lesions.
